# Microscopy of a Deciduous Tooth. Ossified Pulp

**Published:** 1870-09

**Authors:** S. P. Cutler


THE
### AMERICAN JOURNAL
OF
### DENTAL SCIENCE.

Vol. IV. THIRD SERIES--SEPTEMBER, 1870. No. 5.

### ARTICLE I.

### *Microscopy of a Deciduous Tooth. Ossified Pulp.*

By Prof. S. P. Cutler, M.D. D.D.S.

A few days since I extracted a lower cuspid tooth for a girl about 13 years old, about one third of the fang absorbed away, the permanent successor coming up rather on the inside, tooth quite firm, not decayed at all, was not painful, had caused no trouble.

The end of the absorbed fang presented the usual appearance only a little inflamed. I put it into alcohol as usual when I wish to prepare specimens of pulp.

On cracking it in a vice, though not my usual custom, I found it solid and difficult to crush. On examination of the tooth to my great surprise I found no nerve canal as usual, instead quite solid bone; in more definite language the nerve cavity was completely ossified to the end of fang, presenting under the microscope the appearance of an ossified permanent tooth. The ossification was complete except a very small spot in the centre containing no soft portion of pulp, as might have been anticipated, only at end of fang where there was the remains of a portion of soft pulp though I could not find any signs of it in the tooth.

The line of union throughout between primary and secondary was perfect, there being no point of separation even from the effects of partial crushing in the vice, or shrinkage after drying, which is sometimes the case in similar specimens. This secondary formation was more chalky in appearance than usual, not the peculiar transparency characteristic of such secondary dentine in general.

This was not a case of nodular pulp, neither was it a case of *exo dentasis internus*, or gradual occlusion, as occurs from wearing away of crown by mastication or by old age; but a case different from either, a case of complete ossification of pulp with perfect union to interior pulp walls extending to end of absorbed fang. Such extensive ossification of pulp seldom occurs, only in extreme old age, or in cases where there has been extensive absorption of alveolus and irritation as may sometimes be seen. The cause of this unusual formation must have been the slight irritation caused by the developemental encroachment of the future successor and the fine attachments of the deciduous; though this latter is a common occurrence, witnessed daily by dentists, and nothing unusual.

There must be some peculiarity of temperament in this case not readily understood. My attention was not called to this subject at the time of extraction. It is a question how much farther fang absorption would have extended had the tooth been left to nature's processes; if any farther at all the process must have been much retarded as a double barrier would have been presented. The opposite tooth was also extracted and presented about the same appearance but was not examined, this tooth might or might not have been in a similar condition; I regret exceedingly I did not save that tooth for examination also.

Hardened or nodular pulps are a very common occurrence in all classes of teeth, resulting from decays or injuries. This variety of secondary dentine is often met with in young subjects when decays progress rapidly, and is not uncommon in old age. We sometimes notice nodulis in deciduous teeth though not so common.

The most extraordinary feature in this case is the peculiarity of the secondary formation; instead of secondary dentine, as usual, there was only a portion of the pulp presenting the usual appearance it being that of the crown which shows the fibrillar structure, together with almost true or normal lacunæ and canaliculi, only less clearly marked, some of them being very large and irregular in shape, appearing as dark bodies not transmitting as much light as true bone, more corpuscles, others and the most numerous portion nearly resemble true bone, more especially in the fang portion where the fibrillar arrangement is almost entirely wanting, and more completely so near the end of fang. In the fang portion the differentiation of soft pulp into true bone corpuscles is quite complete, the fibrils mainly transformed. In some portions of the crown cavity the continuations of dentinal tubuli into the secondary formation is quite distinct, as in cases of permanent teeth in other portions quite indistinct and obliterated. I have never yet seen a case of nodular dentine or secondary dentine containing true bone structure before this one, and I had entertained the idea from numerous observations that such could not or did not occur, but now I have facts to the contrary in this single case. Now the difficulty in accounting for the transformation appears to be almost unaccountable. I will endeavor to give a solution of the problem: In the formation of either primary or secondary bone, there has to be provided a true bone matrix or cartillage, with always true cartillage corpuscles or cells in all primary bone cartillage not always necessarily the case in secondary, especially in the repair of fractures where adjustment had been perfect.

Bone is a very low order of vitalized structure, the teeth on the contrary very high in some respects especially that of sensibility; even the cement also, that tissue being a sort of hybrid of bone and dentine possessing attributes of both. In order to have true bone there must be a bone matrix improvised in some way, otherwise bone could not be formed when bone cartillage transformation takes place, the original cartillage structure entirely disappears no trace or vestige remains, the change being very striking. There is no apparent communication from one cartillage cell to another, only a homogeneous structure, In bone there are communications by canaliculi from lacunæ to lacunæ, the lacunæ probably occupying the original site of the cartillage cells, and the canals formed during differentiation are the connective canaliculi.

We find in the tooth pulp the elements of dental transformation by infiltration of lime salts into pulp structure, solidifying all but nerve fibrils which, from their actual resistance, reject lime encroachments so long as their neural connection remains intact. But should this connection be cut offin any way, then even the fibrils themselves might undergo this transformation, never without.

In the case above named we find portions where the fibrillar arrangements remains intact, especially in the crown where we may imagine the process to have commenced and before the fang absorption had progressed, for afterwards we may suppose the fibrils to have been more or less completely cut off by absorption at the point of contact with permanent encroacher and at the same time the blood vessels remaining more or less intact carrying lime salts into the partially now resistant pulp ending as the process advanced into some true bone formation. There are appearances of Haversian rods in several places, though not well established by characteristic concentric layers, as in true bone we could not expect this in such cases. We do not find any structure in the soft pulp resembling bone or any other cartillage structure, instead we find the neural fibrillar structure, also blood vessels and a cell structure fill with some oliaginous matters of a volatile nature, probably phosphated fat, though this fact has not yet been fully determined by chemical analysis The pulp cells and even blood vessels suffer differentiation from soft pulp into secondary dentine, by replacement of the cell contents by lime salts, the cell structure remaining intact though not recognisable clearly under the microscope, the fibrils occupying the secondary tubuli, and thus formed retaining to a greater or lesser degree their their normal sentient vitality sometime giving trouble from their sensibility by extent of occupancy by earthy salts, their tubular extent being in such cases abnormal or more extensive the fibrils having an abnormal extent of bone structure, hence abnormal resistance to overcome, in consequence producing disturbance and pain, the result from irregular neural transmission of nerve force to the brain.

---

